# Supplementary figures and images for: The Efficacy of Radiotherapy without Surgery for External Auditory Canal Squamous Cell Carcinoma
Source: J Clin Med. 2022 Oct 6;11(19):5905. doi: 10.3390/jcm11195905 (PMC9571158; doi:10.3390/jcm11195905)

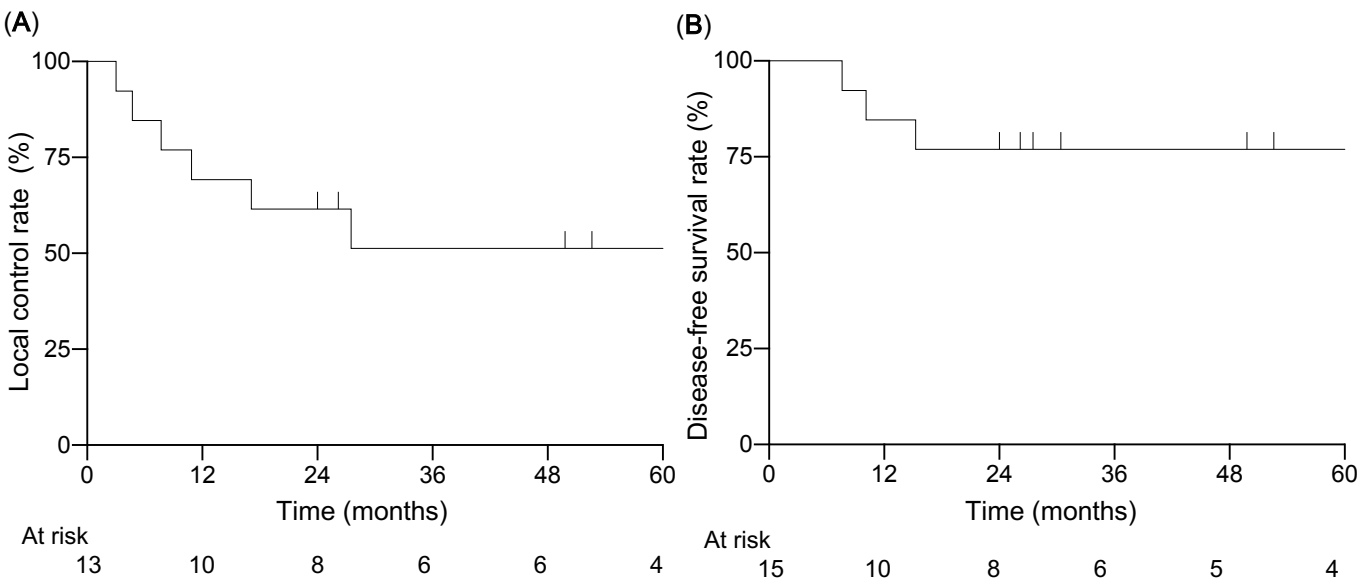

**Supplementary Figure S1.** Kaplan–Meier curves for T2-4 **(A)** local control rate, **(B)** overall survival rate.

Supplement: Supplementary file 1 [file jcm-11-05905-s001.zip › jcm-1921348-supplementary.pdf]
